# Case report and systematic review of mesenteric artery by-pass for non-atherosclerotic mesenteric vascular disease

**DOI:** 10.1016/j.ijscr.2020.04.033

**Published:** 2020-05-11

**Authors:** Wajiha Zahra

**Affiliations:** Doncaster Royal Infirmary, Thorne Road, Doncaster DN2 5LT, United Kingdom

**Keywords:** Mesenteric ischemia, Post-prandial angina, Antiphospholipid syndrome

## Abstract

**Introduction:**

Mesenteric artery re-vascularization using bypass or angioplasty is a standard of care in atherosclerotic mesenteric vascular disease. However, there are no certain guidelines for the management of non-atherosclerotic thrombophilic mesenteric vessel disease other than anti-coagulation.

**Case report:**

We are reporting a case of a 36 years old lady with post-prandial angina and weight loss on the background of anti-phospholipid syndrome having tripple mesentric vessel occlusive disease who underwent 2 vessel aorto-mesenteric bypass. During the post-op course, patient had laparotomy with diversion ileostomy for ischemic perforation of the bowel. At present, after 6 weeks patient is recovering well with improved appetite and resolution of symptoms.

**Discussion:**

There are very few cases reported with acute on chronic mesenteric ischemia in England. Nikolas Melas et al. reported similar history in a 57 yrs old Caucasian female. Morbi AH2 highlights timely diagnosis of acute mesmeric ischemia in a 53 yrs old.

**Conclusion:**

This case report is unique in a way that combined mesenteric ischemia and APS is a rare combination in a 36 years old.

Mesenteric artery bypass is one of the possible solutions to thrombophilic mesenteric disease, however larger studies with longer follow up are needed.

## Highlights

• Few cases reported in England.
• CMI and antiphospholipid syndrome is a rare combination in a 36 years old.
• Endovascular options are not feasible due to 3 vessel occlusion at origin.

## 1 Introduction

Chronic mesenteric ischaemia (CMI) is defined as ischaemic symptoms caused by insufficient blood supply to the gastrointestinal tract in at least 3 months. The typical presentation includes postprandial pain, weight loss resulting from fear of eating, or unexplained diarrhoea [1]. Mesenteric Ischemia includes inadequate blood supply, inflammatory injury and eventually necrosis of the bowel wall. The disease can be divided into acute and chronic MI (CMI), with the first being subdivided into four categories [2]. Therefore, acute MI (AMI) can occur as a result of arterial embolism, arterial thrombosis, mesenteric venous thrombosis (MVT) and non-occlusive causes (NOMI), such as hypo-perfusion due to low cardiac output or mesenteric arterial vasoconstriction [3]. Bowel damage is in proportion to the mesenteric blood flow decrease and may vary from minimum lesions, due to reversible ischemia, to transmural injury, with subsequent necrosis and perforation [4]. CMI is associated to diffuse atherosclerotic disease in more than 95% of cases, with all major mesenteric arteries presenting stenosis or occlusion [5]. Patients with conditions that predispose them to atherosclerosis, such as hypertension, diabetes mellitus, and hypercholesterolemia, are at increased risk for CMI [6]. Antiphospholipid syndrome (APS) is a rare disease characterised by venous and/or arterial thrombosis, pregnancy complications and the presence of specific autoantibodies called antiphospholipid antibodies [7] which can predispose CMI.

## 2 Case report

We report case of a young 36 years old English female as per SCARE [8] criteria, who presented to emergency department with 4 months history of worsening abdominal pain, diarrhoea and vomiting. Her pain gets worse after food and she reported loss of 2 stones during this time. Apart from IBS, she had 3 miscarriages in the past. She was under investigation for antiphospholipid syndrome by haematology team and had positive anticardiolipin antibodies. She reported no other co-morbidities. She quit smoking a year ago.

On admission, her inflammatory markers were significantly high. CT scan showed occlusion of Celiac Trunk and SMA. IMA could not be identified. Very minimal enhancement of small bowel loop effecting the distal ileum in the pelvis concerning for ischemic segment.

Despite proactive implementation of treatment dose heparin, aspirin and statin her pain didn’t improve and it was decided by vascular MDT to perform surgery. She underwent emergency aorto-common hepatic and aorto-SMA bypass graft done by team of experienced vascular consultants. After 1st surgery, she underwent explorative laparotomy which showed an intestinal perforation and had resection of 30 cm ischemic bowel. After the procedure, she stayed on treatment dose IV heparin. 5 days post- bowel resection she dropped her Hb and had to undergo 3rd explorative laparotomy for Pelvic hematoma, which was evacuated, cavity was washed and vac dressing applied. In 3rd exploration, anastomosis was intact and bowel was healthy. She had a long hospital stay due to nutrition requirements but recovered well and was discharged after 28 days.

## 3 Discussion

There are very few cases reported in England with acute on chronic mesenteric ischemia. Nikolas Melas et al. reported similar history in a 57 years old Caucasian female, who presented with extreme weight loss, postprandial abdominal pain and diarrhoea. She had CT, SMA occlusion and had bilateral renal arteries stenosis. The occluded vessels were both stented after an angioplasty and ischemic segment was resected. Patient was discharged on lifelong warfarin [9]. Morbi AH and Nordon IM highlights the importance of timely diagnosis and acute mesenteric ischemia in a 53 year old female. Her CT scan showed small bowel ischaemia, chronic occlusion of the coeliac axis, and a long acute-on-chronic occlusion of the superior mesenteric artery (SMA). The length and morphology of the SMA occlusion precluded endovascular treatment. Patient had aorto-SMA bypass with small bowel resection [10]. Salaun et al. from Netherlands reported stenosis of SMA and left renal artery and thrombosis of coeliac trunk in a 33 years old presenting with abdominal angina and hypertension. Patient had angioplasty and stenting of SMA and left renal artery and was discharged on anticoagulant and antiplatelet [11]. European Society of Vascular Surgery (ESVS) guidelines on mesenteric ischemia suggests revascularisation is indicated in patients who develop symptoms of CMI. There is no role for a conservative approach with long-term chronic parenteral nutrition and non-interventional therapy. In fact, excessive delays in proceeding with definitive revascularisation or use of parenteral nutrition alone have been associated with clinical deterioration, bowel infarction, and risk of sepsis from catheter related complications. Surgery also may be preferable in patients who have non-atherosclerotic causes such as vasculitis, neurofibromatosis, and mid-aortic syndrome [1].

## 4 Conclusion

Our case report is unique in a way that combined mesenteric ischemia and antiphospholipid is a rare combination in a 36 years old. In a young patient with APS conservative management was an option but she ultimately needed surgery. Not many such cases have been reported in Britain before. It also emphasizes the timely diagnosis and management of antiphospholipid syndrome. It still needs to be seen whether patients diagnosed with APS needs surveillance for bowel ischemia or early intervention with anticoagulants would have made any difference.

## Declaration of Competing Interest

Nil.

## Funding

NA.

## Ethical approval

Not applicable.

## Consent

Written informed consent was obtained from the patient for publication of this case report.

## Author contribution

Concept, Discussion and literature review done by Wajiha Zahra.

## Registration of research studies

NA.

## Guarantor

Wajiha Zahra.

## Provenance and peer review

Not commissioned, externally peer-reviewed.
